# Supplementary material for: Long Circulating RNAs Packaged in Extracellular Vesicles: Prospects for Improved Risk Assessment in Childhood B-Cell Acute Lymphoblastic Leukemia
Source: Int J Mol Sci. 2025 Apr 22;26(9):3956. doi: 10.3390/ijms26093956 (PMC12071302; doi:10.3390/ijms26093956)
Supplement: Supplementary file 1 [file ijms-26-03956-s001.zip › Poncelet_Supplemental_Data_IJMS_09042025.pdf]

Supplemental data

Bioinformatics Analyses

Custom transcripts annotation construction

A custom annotation of the genome was created to quantify a wide variety of coding and non-coding linear RNAs, specific fusion transcripts and circRNAs using a single program (Salmon 1.9.0).

<u>Linear RNAs:</u> Based on previous work,[1] we created a non-redundant merge of the publicly available GENCODE V29 and LNCipedia 5.2[2] annotations.

<u>Circular RNAs:</u> Unlike most RNA molecules, circRNAs form closed loops by joining the 3’ and 5’ ends of the RNA sequence through a process called backsplicing.[3] They are commonly studied apart from other transcripts in RNAseq analysis since most tools currently available for their detection and quantification report values based on backsplice junction (BSJ) counts.[4] Recently, model-based strategy has been implemented to quantify circRNAs,[5, 6] reporting count values comparable with linear transcripts. No study yet has used such an approach to report proportions of circular transcripts in RNAseq data compared to linear transcript. All samples included in this study were aligned to the GRCh38 human genome reference sequence using both STAR 2.7.8[7] and BWA 0.7.17[8] for downstream analysis with CIRCexplorer 2.3.3[9] and CIRI 2.0.6[10] for circRNAs detection and annotation. A non-redundant merge of all discovered circRNAs was output in a single BED format file and converted into a pseudo-linear reference in FASTA format using a modified version of sailfish-cir 0.11[6] omitting the index build and quantification steps.

<u>Fusion transcripts:</u> Similarly, fusion genes expressed in *ETV6::RUNX1*$^{+}$ and *TCF3::PBX1*$^{+}$ tumors are quantified using special tools detecting chromosomal rearrangements and counting fusion breakpoints. Model-based strategy has not been implemented for fusion transcripts quantification. Total RNAseq data from patient’s tumor as well as whole cell extracts from the REH and 697 cell lines were analysed using

STAR-Fusion 1.11.0[11] and arriba 1.2.0[12] for fusion events detection and breakpoints identification. The collected data was used to build a custom FASTA reference of the observed variants of the *ETV6::RUNX1*, and *TCF3::PBX1* fusion transcripts present in the dataset.

All three annotations were merged into a single FASTA file and concatenated with the GRCh38 reference genome before building a fully decoy aware Salmon index.

Correlation and ontology analyses

Genes expressed in tumor samples of 12 patients from the *ETV6::RUNX1*$^+$ subtype, 12 patients from the *TCF3::PBX1*$^+$ subtype and 3 blood-cord derived B-cell controls were ranked according to expression variance across samples using DESeq2. Two distinct datasets were constructed, one with patients and controls and the other with patients only including the top 5000 most variable genes and the 27 candidate genes identified as potential biomarkers in exosomes. Both were used as input for weighted gene co-expression network analysis (WCGNA) using the WGCNA 1.72-1 R package[13] and the following parameters: power = 10, minModuleSize = 100, mergeCutHeight = 0.25. The resulting modules were assessed for their correlation between B-ALL vs Controls (CD10$^+$/CD19$^+$ B-Cells purified from the cord blood of healthy donors) and *ETV6::RUNX1*$^+$ vs *TCF3-PBX1*$^+$ subtypes. Genes included in the modules most significantly correlated with those traits were used as input for Active-subnetwork-oriented Pathway Enrichment Analysis with the pathfinder 2.1.0 R package[14] and the gene-sets GO-ALL or KEGG. From the same RNA-seq dataset, we assessed correlation between the expression of the identified 47 putative RNA biomarker candidates with the expression of all protein coding genes from the 24 tumors using the fgsea 1.24.0 R package[15] to identify significant enrichment or depletion of 50 predefined gene-sets from the H: hallmark gene sets MSigDB collection.[16, 17] The heatmap of aggregated enrichment scores for all candidates was drawn using the ComplexHeatmap 3.17 R package.[18]

Supplemental Tables

The following supplementary tables are joined as a separate Excel file containing a README tab precising the data structure.

Supplemental Table S1: Genes expressed in RNAseq data from REH and 697 cell lines, ETV6::RUNX1 and TCF3::PBX1 tumors and derived IM-sEVs

Supplemental Table S2: Expression levels of the 102 potential biomarker candidates in RNA-seq data from blood IM-sEVs of B-ALL patients and Healthy controls

Supplemental Table S3: Expression levels of the 102 potential biomarker candidates in RNA-seq data from tumor samples and controls

Supplemental Table S4: Potential biomarker candidates identified in modules correlated with disease status or subtype using WGCNA

Supplemental Table S5: Gene specific primers for RT-qPCR validation of RNA-seq results

Supplemental Figures

Figure S1

A

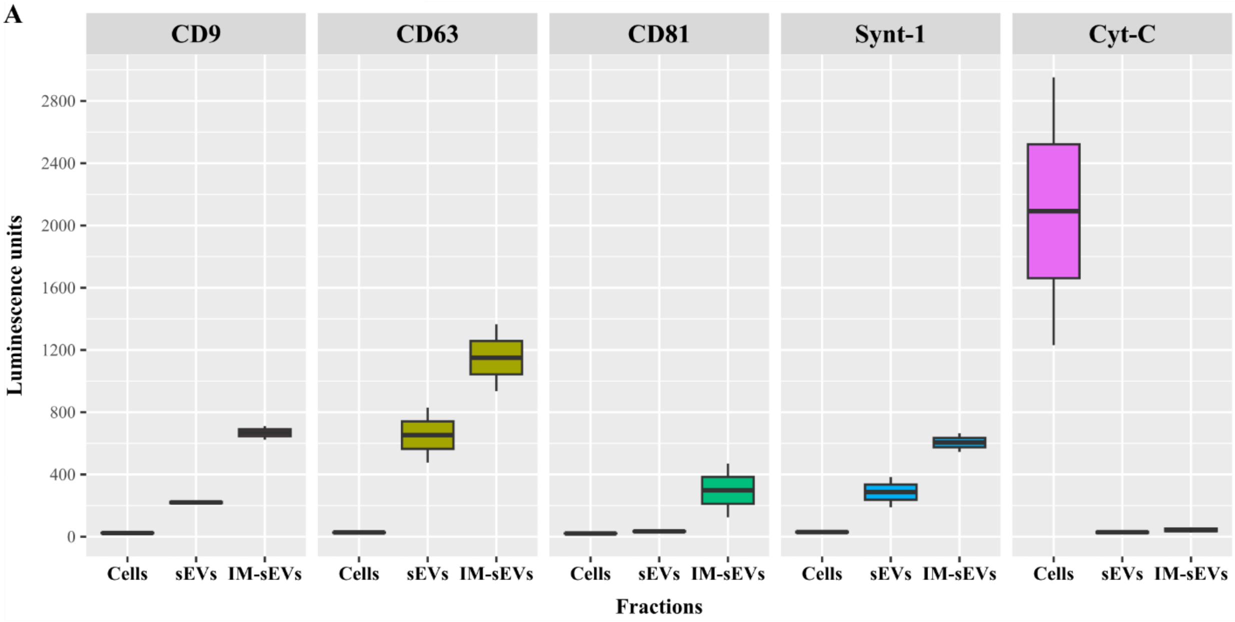

B

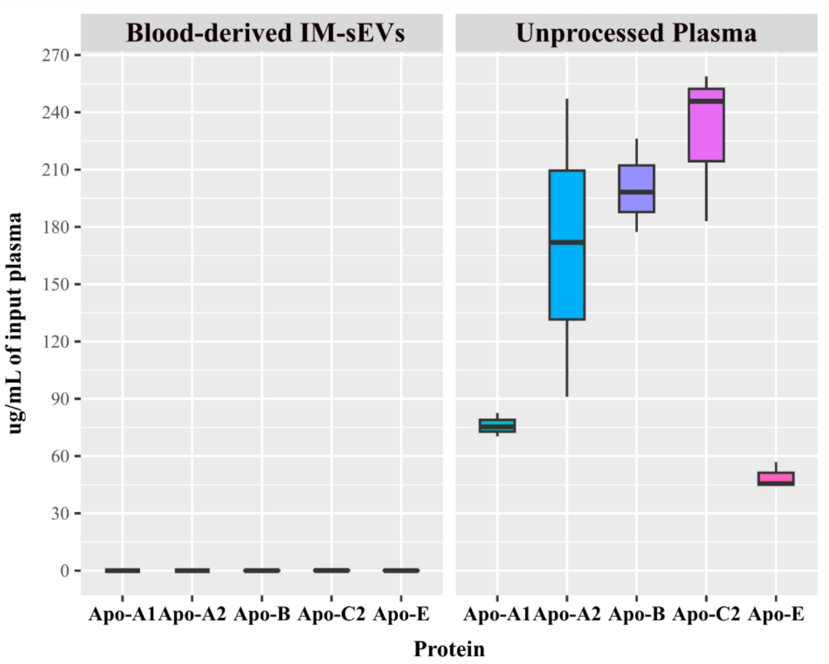

Supplemental Figure S1: (A) Boxplot showing the concentrations in whole cell extracts and two EV populations (sEVs and IM-sEVs) of four protein markers known to be enriched in EVs (CD9,CD63,CD81 ans Synt-1) and Cyt-C as a protein marker of contamination from mitochondria. (B) Boxplot showing the

concentrations in Blood-derived IM-sEVs VS Unprocessed Plasma samples used as input of a panel of 5 lipoproteins (Apo-A1, Apo-A2, Apo-B, Apo-C2 and Apo-E).

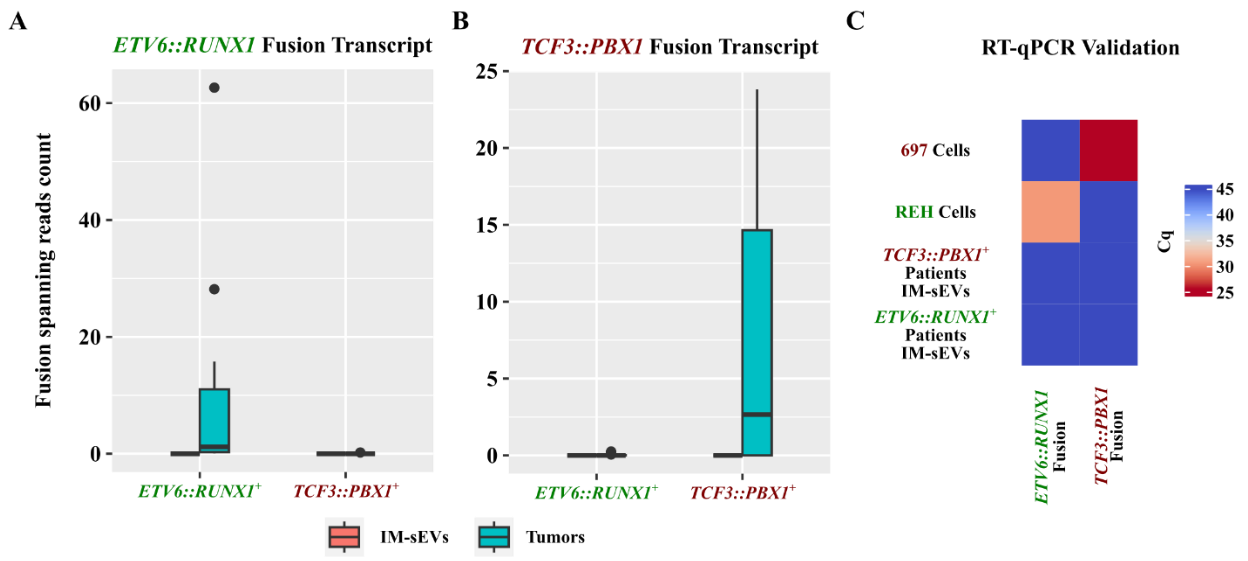

Supplemental Figure S2: Boxplot showing the number of fusion transcript spanning reads detected in total RNA-seq data from tumors (24) and IM-sEVs (10/24) of *ETV6::RUNX1*$^{+}$ (12/24) and *TCF3::PBX1*$^{+}$ (12/24) patients. (A) *ETV6::RUNX1*$^{+}$ fusion transcript spanning reads and (B) *TCF3::PBX1*$^{+}$ fusion transcript spanning reads. (C) Heatmap showing the expression levels reported as Cq values for *ETV6::RUNX1* and *TCF3::PBX1* fusion genes in Patient derived IM-sEVs with 697 and REH whole-cell extracts as positive controls.

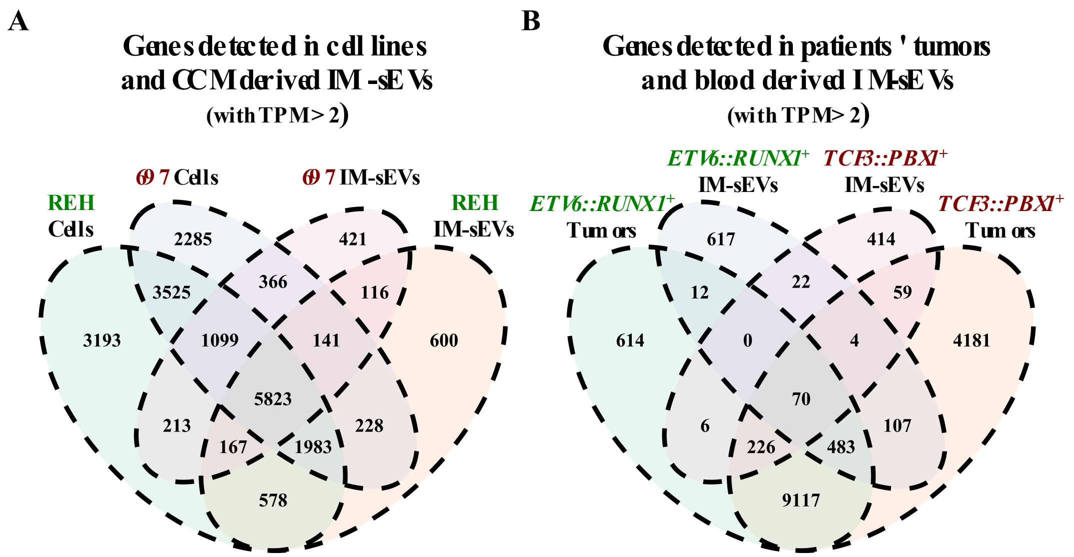

Supplemental Figure S3: Characterization of the long transcriptome in circulating IM-sEVs of childhood B-ALL patients and cell lines. (A) Four-way Venn diagram showing genes detected with a TPM > 2 threshold in RNA-seq data from whole cell extract and IM-sEVs purified from CCM of the REH (*ETV6::RUNX1*$^{+}$) and 697 (*TCF3::PBX1*$^{+}$) cell lines, (B) Four-way Venn diagram showing genes detected with a TPM > 2 threshold in RNA-seq data from whole tumors (n = 24) and IM-sEVs purified from the peripheral blood of *ETV6::RUNX1*$^{+}$ and *TCF3::PBX1*$^{+}$ patients (n = 10).

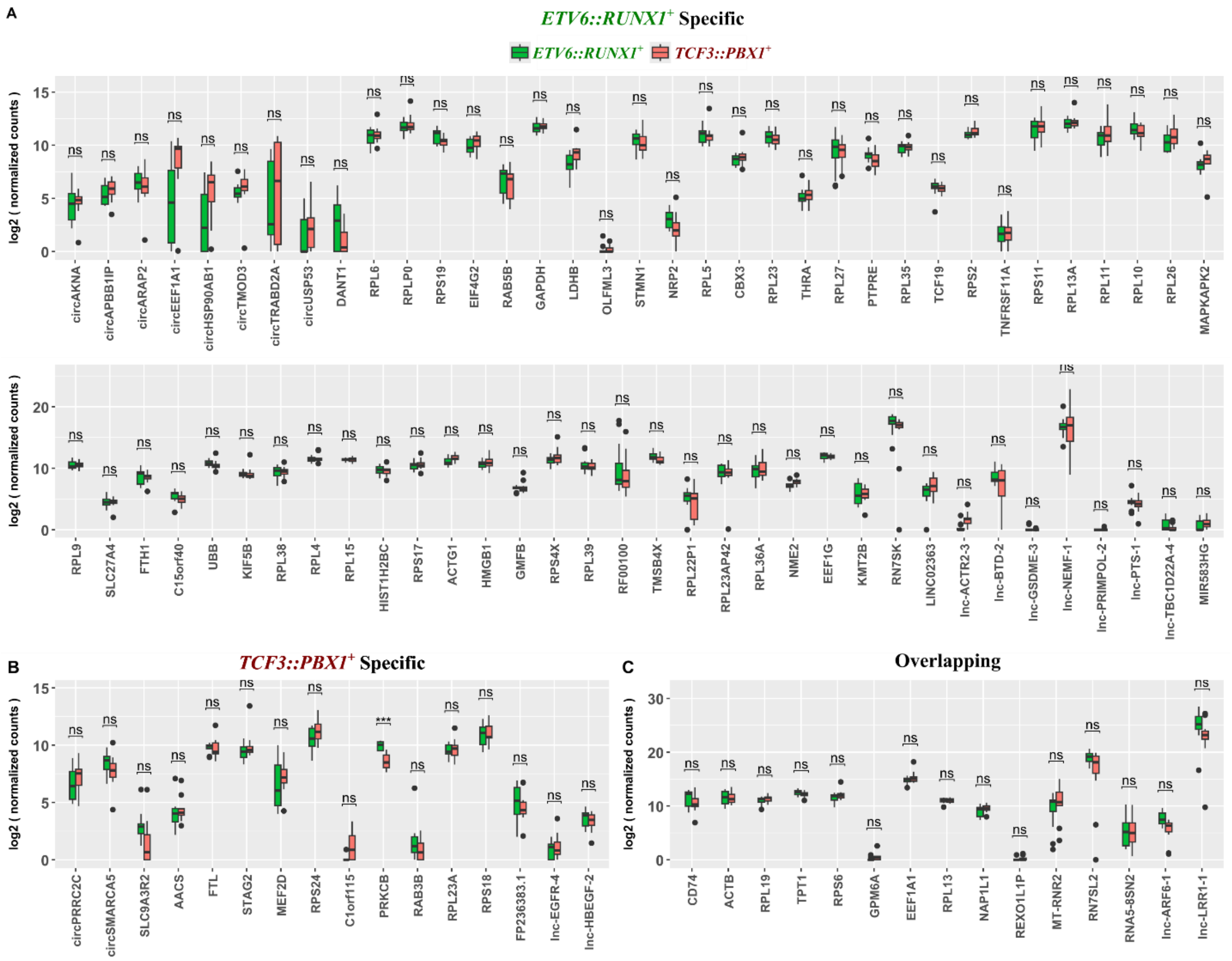

Supplemental Figure S4: Boxplots showing a comparison of expression levels in tumoral RNA of 12 *ETV6::RUNX1*$^{+}$ versus 12 *TCF3::PBX1*$^{+}$ patients for the 102 potential childhood B-ALL biomarker candidates identified in IM-sEVs. The expression levels are reported as log2 transformed RNAseq raw read counts normalized using DESeq2. (A) *ETV6::RUNX1*$^{+}$ specific candidates. (B) *TCF3::PBX1*$^{+}$ specific candidates. (C) Candidates found in IM-sEVs from both subtypes; ns *p*-value > 0.05; *** *p*-value < 0.001.

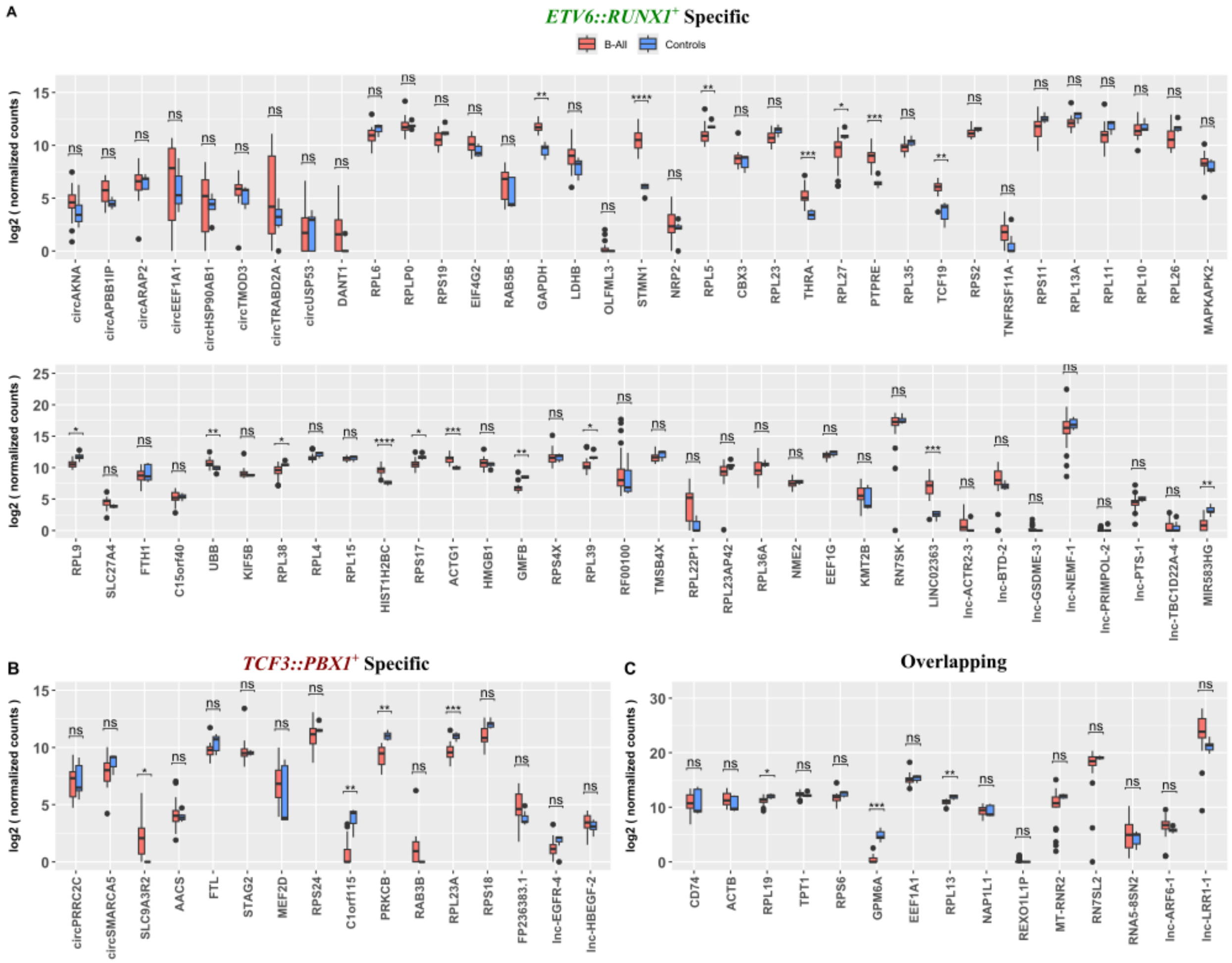

Supplemental Figure S5: Boxplots showing a comparison of expression levels in tumoral RNA of 24 B-ALL patients (12 *ETV6::RUNX1*$^+$ and 12 *TCF3::PBX1*$^+$) versus 3 CD10$^+$/CD19$^+$ B-Cell isolated from healthy cord blood as controls for the 102 potential childhood B-ALL biomarker candidates identified in IM-sEVs. The expression levels are reported as log2 transformed RNAseq raw read counts normalized using DESeq2. (A) *ETV6::RUNX1*$^+$ specific candidates. (B) *TCF3::PBX1*$^+$ specific candidates. (C) Candidates found in IM-sEVs from both subtypes; ns p-value > 0.05; * p-value < 0.05; ** p-value < 0.01; *** p-value < 0.001.

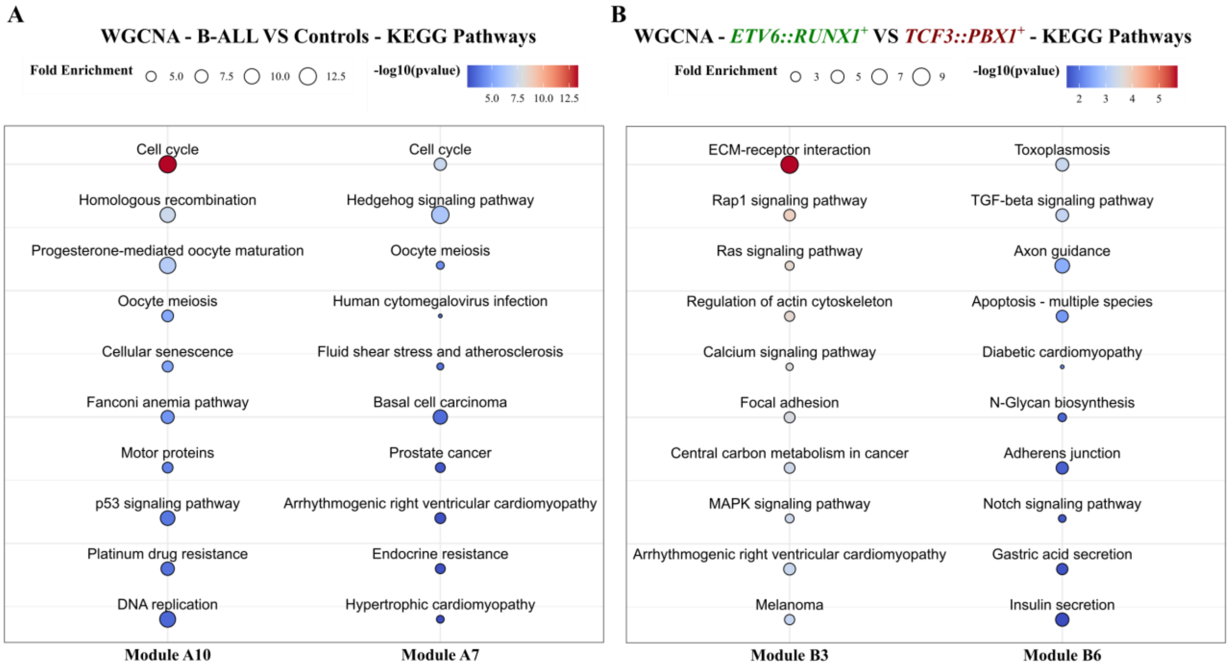

Supplemental Figure S6: Bubble plot showing the top 10 KEGG pathways enriched in (A) the A6 and A7 modules correlated with childhood B-ALL and (B) the B3 and B8 modules, respectively correlated with the *ETV6::RUNX1*$^{+}$ and *TCF3::PBX1*$^{+}$ disease subtypes.

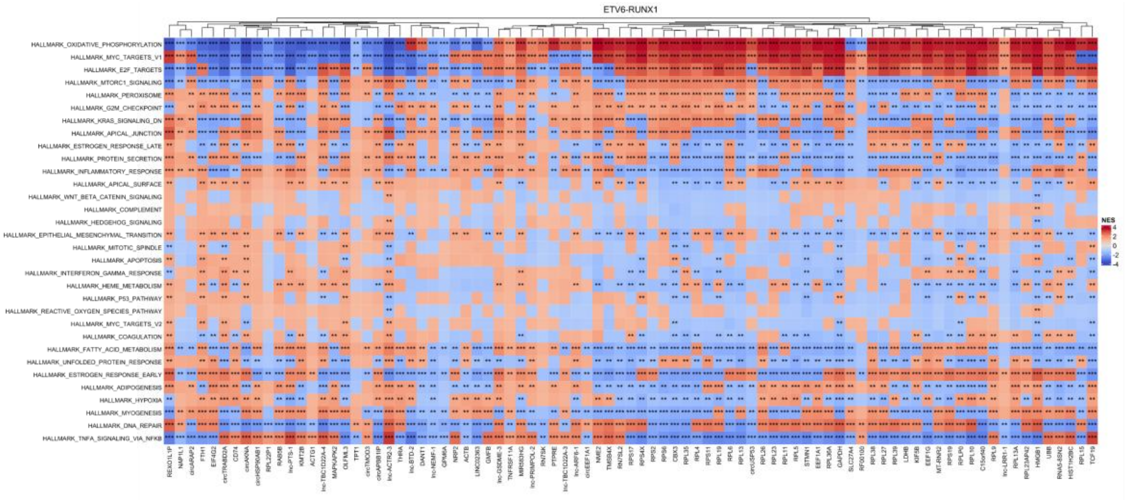

Supplemental Figure S7: Detailed heatmap depicting the correlation between the expression of the 102 identified childhood B-ALL RNA biomarker candidates and 33/50 MSigDB hallmark gene-sets for the *ETV6::RUNX1*$^{+}$ subtype only. Correlations assessed by the FGSEA package using an adaptive multi-level split Monte Carlo scheme; * *p*-value < 0.05; ** *p*-value < 0.01; *** *p*-value < 0.001.

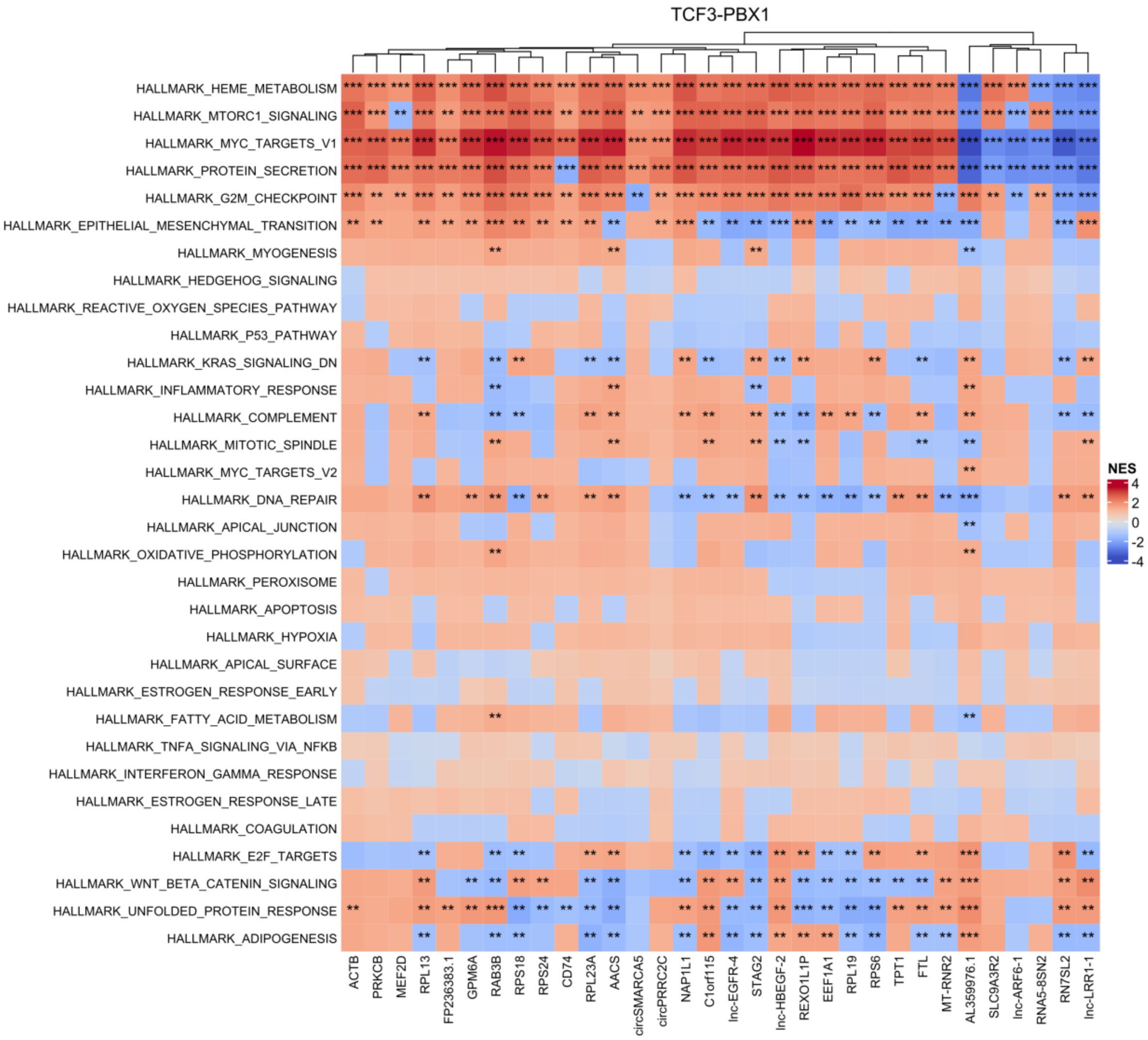

Supplemental Figure S8: Detailed heatmap depicting the correlation between the expression of the 102 identified childhood B-ALL RNA biomarker candidates and 33/50 MSigDB hallmark gene-sets for the *TCF3::PBX1*⁺ subtype only. Correlations assessed by the FGSEA package using an adaptive multi-level split Monte Carlo scheme; * *p*-value < 0.05; ** *p*-value < 0.01; *** *p*-value < 0.001.

References

1. Rodosthenous, R. S.; Hutchins, E.; Reiman, R.; Yeri, A. S.; Srinivasan, S.; Whitsett, T. G.; Ghiran, I.; Silverman, M. G.; Laurent, L. C.; Van Keuren-Jensen, K.; Das, S., Profiling Extracellular Long RNA Transcriptome in Human Plasma and Extracellular Vesicles for Biomarker Discovery. *iScience* **2020,** 23, (6), 101182.
2. Volders, P.-J.; Helsens, K.; Wang, X.; Menten, B.; Martens, L.; Gevaert, K.; Vandesompele, J.; Mestdagh, P., LNCipedia: a database for annotated human lncRNA transcript sequences and structures. *Nucleic Acids Research* **2012,** 41, (D1), D246-D251.
3. Jeck, W. R.; Sharpless, N. E., Detecting and characterizing circular RNAs. *Nature Biotechnology* **2014,** 32, (5), 453-461.
4. Vromman, M.; Anckaert, J.; Bortoluzzi, S.; Buratin, A.; Chen, C.-Y.; Chu, Q.; Chuang, T.-J.; Dehghannasiri, R.; Dieterich, C.; Dong, X.; Flicek, P.; Gaffo, E.; Gu, W.; He, C.; Hoffmann, S.; Izuogu, O.; Jackson, M. S.; Jakobi, T.; Lai, E. C.; Nuytens, J.; Salzman, J.; Santibanez-Koref, M.; Stadler, P.; Thas, O.; Vanden Eynde, E.; Verniers, K.; Wen, G.; Westholm, J.; Yang, L.; Ye, C.-Y.; Yigit, N.; Yuan, G.-H.; Zhang, J.; Zhao, F.; Vandesompele, J.; Volders, P.-J., Large-scale benchmarking of circRNA detection tools reveals large differences in sensitivity but not in precision. *Nature Methods* **2023**.
5. Wen, G.; Li, M.; Li, F.; Yang, Z.; Zhou, T.; Gu, W., AQUARIUM: accurate quantification of circular isoforms using model-based strategy. *Bioinformatics* **2021,** 37, (24), 4879-4881.
6. Li, M.; Xie, X.; Zhou, J.; Sheng, M.; Yin, X.; Ko, E.-A.; Zhou, T.; Gu, W., Quantifying circular RNA expression from RNA-seq data using model-based framework. *Bioinformatics* **2017,** 33, (14), 2131-2139.
7. Dobin, A.; Davis, C. A.; Schlesinger, F.; Drenkow, J.; Zaleski, C.; Jha, S.; Batut, P.; Chaisson, M.; Gingeras, T. R., STAR: ultrafast universal RNA-seq aligner. *Bioinformatics* **2013,** 29, (1), 15-21.
8. Li, H.; Durbin, R., Fast and accurate short read alignment with Burrows-Wheeler transform. *Bioinformatics* **2009,** 25, (14), 1754-60.
9. Zhang, X. O.; Dong, R.; Zhang, Y.; Zhang, J. L.; Luo, Z.; Zhang, J.; Chen, L. L.; Yang, L., Diverse alternative back-splicing and alternative splicing landscape of circular RNAs. *Genome Res* **2016,** 26, (9), 1277-87.
10. Gao, Y.; Wang, J.; Zhao, F., CIRI: an efficient and unbiased algorithm for de novo circular RNA identification. *Genome Biology* **2015,** 16, (1), 4.
11. Haas, B. J.; Dobin, A.; Stransky, N.; Li, B.; Yang, X.; Tickle, T.; Bankapur, A.; Ganote, C.; Doak, T. G.; Pochet, N.; Sun, J.; Wu, C. J.; Gingeras, T. R.; Regev, A., STAR-Fusion: Fast and Accurate Fusion Transcript Detection from RNA-Seq. *bioRxiv* **2017**, 120295.
12. Uhrig, S.; Ellermann, J.; Walther, T.; Burkhardt, P.; Fröhlich, M.; Hutter, B.; Toprak, U. H.; Neumann, O.; Stenzinger, A.; Scholl, C.; Fröhling, S.; Brors, B., Accurate and efficient detection of gene fusions from RNA sequencing data. *Genome Res* **2021,** 31, (3), 448-460.
13. Langfelder, P.; Horvath, S., WGCNA: an R package for weighted correlation network analysis. *BMC Bioinformatics* **2008,** 9, (1), 559.
14. Ulgen, E.; Ozisik, O.; Sezerman, O. U., pathfindR: An R Package for Comprehensive Identification of Enriched Pathways in Omics Data Through Active Subnetworks. *Front Genet* **2019,** 10, 858.
15. Korotkevich, G.; Sukhov, V.; Budin, N.; Shpak, B.; Artyomov, M. N.; Sergushichev, A., Fast gene set enrichment analysis. *bioRxiv* **2021**, 060012.
16. Subramanian, A.; Tamayo, P.; Mootha, V. K.; Mukherjee, S.; Ebert, B. L.; Gillette, M. A.; Paulovich, A.; Pomeroy, S. L.; Golub, T. R.; Lander, E. S.; Mesirov, J. P., Gene set enrichment

analysis: A knowledge-based approach for interpreting genome-wide expression profiles. *Proceedings of the National Academy of Sciences* **2005,** 102, (43), 15545-15550.
17. Liberzon, A.; Birger, C.; Thorvaldsdóttir, H.; Ghandi, M.; Mesirov, J. P.; Tamayo, P., The Molecular Signatures Database (MSigDB) hallmark gene set collection. *Cell Syst* **2015,** 1, (6), 417-425.
18. Gu, Z., Complex heatmap visualization. *iMeta* **2022,** 1, (3), e43.
